# Energy Cost of Walking in Relation to Impairment and Functional Independence After Stroke: The Role of Walking Speed

**DOI:** 10.1002/pri.70287

**Published:** 2026-07-14

**Authors:** Amine Guediri, Maxence Compagnat, Alix Poyet, Jean‐Christophe Daviet, Stéphane Mandigout

**Affiliations:** ^1^ HAVAE, UR 20217 University of Limoges Limoges France; ^2^ Department PMR CHU Limoges Limoges France

**Keywords:** activities of daily living, energy metabolism, stroke, walking

## Abstract

**Background and Purpose:**

Stroke survivors often exhibit reduced walking efficiency, reflected by an increased energy cost of walking (Cw). Although Cw has been associated with walking performance, its relationship with impairment severity and independence in activities of daily living (ADL) remains unclear, particularly across real‐life walking conditions. This study aimed to examine the association between impairment severity and Cw across real‐life walking tasks. A secondary objective was to assess the association between Cw and independence in ADL.

**Methods:**

This secondary analysis used data from a cross‐sectional study including 30 stroke survivors. Participants performed walking tasks under real‐life conditions (overground, irregular surface, slope, and stairs) at self‐selected speeds. Oxygen consumption was measured using portable indirect calorimetry, and Cw was calculated as the ratio of oxygen consumption to walking speed. Impairment severity and independence in ADL were assessed using the NIHSS and the Barthel Index. Associations were analyzed using Spearman's correlations, with additional analyses adjusted for walking speed.

**Results:**

Unadjusted analyses showed significant associations between Cw and impairment severity across overground walking, stair descent, irregular terrain walking, and downhill walking (*ρ* = 0.38–0.59). Cw was also significantly associated with independence in ADL across overground walking, stair descent, irregular terrain walking, uphill, and downhill walking (*ρ* = −0.38 to −0.59), with the strongest associations generally observed during irregular terrain and downhill walking. After adjustment for walking speed, none of these associations remained statistically significant (all *p* > 0.05).

**Discussion:**

Cw was associated with both impairment severity and independence in ADL, but these relationships were largely explained by walking speed. These findings suggest that gait velocity is a central factor linking walking energetics to clinical and functional outcomes after stroke. Limitations include the relatively mild impairment level and the cross‐sectional design.

**Trial Registration:**

ClinicalTrials.gov identifier: NCT05477238

## 1 Introduction

Physical activity (PA) plays a central role in post‐stroke recovery. It contributes to improved cardiovascular health, muscle strength, and gait performance, while also supporting neuroplasticity and functional independence (Billinger et al. 2014; Han et al. 2017; Krakauer and Marshall 2015; MacKay‐Lyons et al. 2019; Pang et al. 2013; Ploughman et al. 2015; Saunders et al. 2020; Zeiler and Krakauer 2013). However, several systematic reviews have shown that most stroke survivors engage in insufficient PA levels to achieve meaningful health benefits (English et al. 2014; Field et al. 2013; Fini et al. 2017). This low PA level is multifactorial, reflecting physical, physiological, psychological, and environmental barriers (Thilarajah et al. 2018; Guediri et al. 2025). Among the different components of PA, walking represents a core activity after stroke (Fini et al. 2022) and largely determines overall daily PA levels (Muren et al. 2008). In this context, increased energy costs directly affect walking performance (Franceschini et al. 2013). Several studies have demonstrated a negative association between the energy cost of walking (Cw)‐defined as oxygen consumption (VO_2_) divided by walking speed‐ and daily step count (Franceschini et al. 2013; Ribeiro et al. 2019). In fact, compared with healthy individuals, stroke survivors exhibit a 1.5‐ to 2‐fold increase in VO_2_ during walking, reflecting elevated metabolic demand, reduced efficiency, and greater susceptibility to fatigue (Polese et al. 2018).

This increased Cw is mainly explained by altered gait parameters, such as reduced walking speed (Polese et al. 2018; Reisman et al. 2009) and step length asymmetry (Awad et al. 2015), as well as physiological mechanisms including autonomic nervous system dysfunction (Billinger et al. 2011), impaired respiratory function (Billinger et al. 2011; Lanini et al. 2003), and reduced muscular oxygen extraction capacity (Billinger et al. 2011; Billinger and Kluding 2009). Beyond these biomechanical and physiological mechanisms, it would also be relevant to determine whether clinical factors can help identify increased Cw after stroke. While stroke‐related impairments are well known to directly affect daily step count (Guediri et al. 2025), it remains unclear whether the severity of these impairments also influences Cw. Previous evidence on this relationship remains limited. Reisman et al. (2009) have examined this relationship, reporting a significant negative correlation between motor impairment severity, assessed by the Fugl‐Meyer score (*r* = −0.65, *p* = 0.006), and Cw. However, this investigation was restricted to treadmill walking, which does not reflect the variety of walking conditions encountered in daily life, such as stair negotiation, uneven surfaces, or slope walking.

Building on this impairment's impact on Cw, it is also important to examine whether increased Cw is associated with independence in daily life. It is well established that increased Cw negatively affects walking (Franceschini et al. 2013), and that walking is a key determinant of independence in activities of daily living (ADL) (Muren et al. 2008). However, evidence regarding the direct relationship between Cw and independence in ADL in stroke survivors remains limited. Compagnat, Mandigout, et al. (2021) reported a negative correlation between the Barthel Index and Cw (*r* = −0.51, *p* < 0.01) during a 6‐min walk test. Nevertheless, this investigation is limited to flat‐ground walking, whereas real‐life mobility often involves more variable and demanding conditions such as stair negotiation, slope walking, and irregular terrain, which represent common community ambulation challenges encountered by stroke survivors. Exploring this relationship across different walking contexts may help determine whether the association between Cw and independence in ADL depends on the walking situation and may provide additional insight beyond conventional flat‐ground walking assessments.

Therefore, this study aims to examine the association between clinical impairment severity and Cw during walking across different real‐life walking tasks. A secondary objective was to examine the association between Cw and independence in ADL across these different walking contexts.

## 2 Method

### 2.1 Study Design and Ethical Approval

This secondary analysis used data from a monocentric, cross‐sectional study conducted at the University Hospital Center of Limoges, in the Department of Physical and Rehabilitation Medicine (Jean Rebeyrol Hospital), from September 2022 to June 2023. The present analysis focused exclusively on the stroke cohort.

Stroke participants were recruited during their inpatient or outpatient rehabilitation. The protocol was approved by the Ouest 1 Ethics Committee on July 12, 2022 (reference: 2022‐A01175‐38) and classified as a Type 2 interventional study according to French Jardé law. The study was registered on ClinicalTrials.gov (NCT05477238). All participants provided written informed consent prior to inclusion.

### 2.2 Eligibility Criteria

Participants were eligible if they were 18 years or older, able to provide informed consent, not under legal protection, and affiliated with the French national health insurance system. Participants had to present a stroke diagnosis confirmed by brain imaging and demonstrate sufficient ambulatory capacity to ascend and descend stairs without physical assistance, corresponding to a modified Functional Ambulation Classification (mFAC) score ≥ 6. Exclusion criteria included any contraindication to PA in accordance with the 2022 French national recommendations (Consultation et prescription médicale d’activité physique à des fins de santé chez l’adulte 2022) (e.g., unstable diabetes or hypertension, angina, intracardiac thrombus), as well as severe cognitive impairment (MoCA < 18).

### 2.3 Clinical and Demographic Data

The severity of neurological impairments was assessed using the National Institutes of Health Stroke Scale (NIHSS). The NIHSS quantifies post‐stroke neurological deficits across several domains including level of consciousness, visual fields, motor strength, coordination, sensory function, and language. The total score ranges from 0 (no deficit) to 42 (severe deficit), with higher scores indicating greater impairment (Brott et al. 1989). Global functional independence was measured using the Barthel Index, a validated evaluation of ADL including feeding, dressing, hygiene, continence, indoor mobility, and stair climbing. Total scores range from 0 to 100, with higher values indicating greater independence (Loewen and Anderson 1990; Mahoney and Barthel 1965). Demographic and clinical variables were also collected, including age, sex, weight, height, stroke type and location, time since stroke, and the use of walking aids.

### 2.4 Equipment

Oxygen consumption during walking tasks was assessed using a portable indirect calorimetry system (Metamax 3B, Cortex Biophysik, Leipzig, Germany). The device consists of a measurement unit and battery module and uses a digital bidirectional turbine to record ventilatory volume, an electrochemical sensor for O_2_, and an infrared analyzer for CO_2_. The Metamax 3B has demonstrated high validity and reliability, particularly during low‐intensity locomotor activities (Macfarlane and Wong 2012). Test–retest reliability is excellent, with ICC values above 0.90 (Vogler et al. 2010) and measurement bias generally below 5% across studies (Compagnat, Daviet, et al. 2021). Gas exchange data were transmitted in real time via telemetry and recorded using MetaSoft software (version 5.0). The calibration of flow and gas analyzers was systematically performed before each test according to manufacturer guidelines.

### 2.5 Protocol

Data collection was conducted over two sessions (indoor and outdoor) scheduled within a maximum interval of 14 days to limit clinical fluctuations related to ongoing recovery. Each session began with a resting VO_2_ measurement of at least 3 min, used as a reference to confirm full recovery between tasks. All walking tasks were performed at the participant's self‐selected comfortable pace. To minimize fatigue‐related bias, task order was partially randomized: during the indoor session, participants were randomly assigned to begin with either overground walking or stair climbing; during the outdoor session, participants were randomly assigned with either irregular‐surface walking or uphill walking. Rest periods were provided between tasks until VO_2_ values returned to baseline. Task onset and termination were marked directly in the Metamax 3B interface to ensure accurate segmentation during analysis.

#### 2.5.1 Indoor Walking Tasks

Overground walking was performed along a 60 m hospital corridor. Participants walked back and forth for at least 3 min, allowing VO_2_ to reach a steady state. Stair ascent involved climbing five floors (110 steps: 14 cm height, 32 cm depth) using a handrail if needed. The task stopped at the top floor or earlier if fatigue occurred. Stair descent was then performed on the same staircase under identical conditions.

#### 2.5.2 Outdoor Walking Tasks

Irregular‐surface walking took place on a grassy, uneven terrain adjacent to the rehabilitation unit (120 m loop). Participants walked for a minimum of 3 min to ensure metabolic stabilization. Uphill walking was performed on a 4% incline within the hospital grounds. Following a 150 m flat warm‐up, participants ascended a 45 m slope for a total of ∼200 m. Downhill walking consisted of retracing the same path in reverse, starting from the top of the incline.

### 2.6 VO_2_ Calculation

Oxygen consumption (VO_2_) was expressed in mL·kg^−1^·min^−1^. Data were exported to Excel with values recorded at 1‐s intervals. For tasks with a partially controlled duration (overground walking and irregular‐surface walking), participants walked for at least 3 min. In these tasks, VO_2_ was averaged from the third minute to the end of the trial, based on the assumption that a steady‐state VO_2_ is typically achieved after 3 min of continuous effort. Steady‐state attainment was visually confirmed on the VO_2_ time curve. For tasks with fixed or shorter durations (stair ascent, stair descent, uphill walking, and downhill walking) performance was constrained by environmental configuration (number of steps, slope length) or participant fatigue. For these tasks, VO_2_ was calculated as the mean value over the final minute, which best reflects the stabilized metabolic demand under these conditions.

### 2.7 Speed Calculation

Walking speed (m·s^−1^) was calculated using the formula:

$$\text{Speed}=\frac{\text{Distance}\;\left(m\right)}{\text{Time}\;\left(s\right)}.$$

For overground and irregular‐surface walking, time was recorded with a stopwatch. For stair ascent/descent, uphill, and downhill walking, time was extracted from the start and end markers recorded using the Metamax 3B system. Distances for outdoor tasks were measured using a measuring wheel. For indoor overground walking, distance was determined from 5 m floor markers. For stair ascent and descent, the distance was estimated using the Pythagorean theorem based on the standardized step dimensions (14 cm height; 32 cm depth). The resulting hypotenuse (∼35.4 cm) was multiplied by the total number of steps climbed or descended and then converted into meters. This approach has been previously applied by Polese et al. (2017) to approximate stair‐walking distance.

### 2.8 Cw Calculation

Cw was expressed in mL·kg^−1^·m^−1^. It was calculated by dividing VO_2_, in mL·kg^−1^·min^−1^ by walking speed expressed in meters per minute (m·min^−1^):

$$\text{Cw}=\frac{VO_{2}}{\text{Speed}}.$$

### 2.9 Statistical Analysis

Data normality was assessed using the Shapiro–Wilk test. A per‐protocol approach was applied; missing values were therefore excluded from the analyses.

Associations between Cw and clinical measures, including impairment severity (NIHSS) and independence in ADL (Barthel Index), were first examined using unadjusted Spearman's rank correlation coefficients, given the ordinal structure of the clinical scales. To explore the influence of walking speed on the observed associations, speed‐adjusted partial Spearman correlations were then computed. Although walking speed is mathematically incorporated into the calculation of Cw, these analyses were performed to explore whether the observed associations persisted beyond the influence of gait velocity alone. Given the exploratory nature of the analyses and the relatively limited sample size, no formal correction for multiple comparisons was applied because, although such corrections may reduce the risk of type I error, they may also substantially decrease statistical power and increase the risk of type II error. Therefore, the reported associations should be considered exploratory and interpreted cautiously.

Statistical significance was set at *p* < 0.05. Correlation strength was interpreted as follows: 0–0.3 = negligible, 0.3–0.5 = low, 0.5–0.7 = moderate, 0.7–0.9 = high, and 0.9–1.0 = very high (Hinkle et al. 2003).

## 3 Results

### 3.1 Participant Characteristics

Thirty stroke survivors were included. Participant characteristics are summarized in Table 1. Overall, neurological impairment was generally mild (NIHSS: median 2 [1–11], IQR 1), while independence in ADL was relatively preserved (Barthel Index: median 100 [65–100], IQR 0).

**TABLE 1:** Participants characteristics (*N* = 30).

Characteristic	Value
Age in years	70.8 [34.1–83.9] (12.9)^a^
Sex	11 women
	19 men
Height in cm	167 ± 8
Weight in kg	71 ± 14.6
BMI	25.4 ± 4.4
Duration between the 2 sessions in days	3.5 [1–14] (6)^a^
Stroke localization	23 hemispheric
	6 cerebellar
	1 superficial middle cerebral artery
Stroke type	23 ischemic
	7 hemorrhagic
Stroke onset in months	3.7 [0.5–182.8] (15.7)^a^
Barthel index	100 [65–100] (0)
NIHSS	2 [1–11] (1)
Walking aid use	9 participants

*Note:* Depending on the distribution, data are expressed as mean ± standard deviation or as median [minimum–maximum] and interquartile range (IQR).

^a^data are not normally distributed.

### 3.2 Walking Speed, VO_2_ and CW Across Walking Tasks

Walking performance varied markedly across tasks (Table 2). Walking speed ranged from 0.3 ± 0.1 m·s^−1^ for stair ascent/descent to 0.9 ± 0.3 m·s^−1^ for outdoor tasks. VO_2_ data from one participant during overground walking and from another participant during irregular terrain walking were excluded because the minimum 3 min duration required to reach a steady state could not be completed due to fatigue. The highest VO_2_ values were observed during stair ascent (mean 16.8 ± 3.7 mL·kg^−1^·min^−1^), while the lowest occurred during stair descent (10 ± 1.8 mL·kg^−1^·min^−1^). Cw was also maximal during stair ascent (1.24 ± 0.52 mL·kg^−1^·m^−1^) and minimal during overground walking (0.24 ± 0.06 mL·kg^−1^·m^−1^).

**TABLE 2:** Walking speed, oxygen consumption (VO_2_), and energy cost of walking (Cw) across all tasks.

Walking tasks	Walking speed (m·s^−1^)	VO_2_ (mL·kg^−1^·min^−1^)	Cw (mL·kg^−1^·m^−1^)
Overground walking	0.9 [0.4–1.3] (0.3)	11.8 [7–16] (2)^a^	0.22 [0.15–0.39] (0.08)^a^
Stair ascent	0.3 [0.1–0.6] (0.1)	16.4 [9–24] (5)	1.13 [0.61–3.32] (0.4)^b^
Stair descent	0.2 [0.04–0.7] (0.2)^b^	10.3 [6–14] (2.3)	0.66 [0.27–3.67] (0.6)^b^
Irregular terrain walking	0.9 [0.1–1.4] (0.3)	12.1 [7–17] (2.1)^a^	0.22 [0.16–0.96] (0.08)^a^,^b^
Uphill walking	0.9 [0.1–1.5] (0.3)	14.1 [9–19] (3)	0.25 [0.2–1.63] (0.09)^b^
Downhill walking	0.9 [0.1–1.6] (0.3)	10.7 [6–14] (2.8)	0.19 [0.13–1.06] (0.08)^b^

*Note:* Data are presented as median [minimum–maximum], and interquartile range (IQR) to ensure consistency of data presentation across variables with heterogeneous distributions.

^a^ Twenty‐nine participants.

^b^ data are not normally distributed.

### 3.3 Associations Between Cw and NIHSS Across Walking Tasks

**FIGURE 1:**
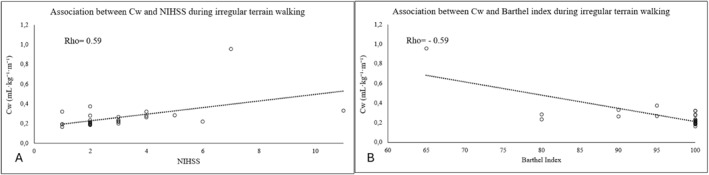
Associations between energy cost of walking (Cw) and clinical scales during irregular terrain walking. (A) Association between Cw and NIHSS. (B) Association between Cw and Barthel Index. Dotted lines are displayed for visualization purposes only and do not imply a fitted linear model.

Unadjusted analyses revealed significant associations between Cw and the NIHSS, across several walking tasks. Low correlations were observed during overground walking (*ρ* = 0.44, *p* = 0.02), stair descent (*ρ* = 0.44, *p* = 0.02), and downhill walking (*ρ* = 0.38, *p* = 0.04), while a moderate correlation was found during irregular terrain walking (*ρ* = 0.59, *p* < 0.001) (Figure 1A). The association observed during stair ascent (*ρ* = 0.37, *p* = 0.05) did not reach statistical significance (Table 3).

**TABLE 3:** Correlations between Cw and clinical scales.

Walking tasks		NIHSS	Barthel index
Overground walking	Unadjusted correlation	***p* = 0.02**	***p* = 0.03**
		***ρ* = 0.44** ^a^	***ρ* = −0.4** ^a^
		**95% CI = 0.08–0.69**	**95% CI = −0.67 to −0.05**
	Speed‐adjusted partial correlation	*p* = 0.09	*p* = 0.41
		*ρ* = 0.32^a^	*ρ* = −0.16^a^
		95% CI = −0.11 to 0.64	95% CI = −0.47 to 0.21
Stair ascent	Unadjusted correlation	*p* = 0.05	*p* = 0.08
		*ρ* = 0.37	*ρ* = −0.33
		95% CI = 0.01–0.64	95% CI = −0.61 to −0.04
	Speed‐adjusted partial correlation	*p* = 0.45	*p* = 0.69
		*ρ* = 0.15	*ρ* = −0.08
		95% CI = −0.33 to 0.59	95% CI = −0.56 to 0.48
Stair descent	Unadjusted correlation	***p* = 0.02**	***p* = 0.04**
		***ρ* = 0.44**	***ρ* = −0.38**
		**95% CI = 0.10–0.69**	**95% CI = −0.65 to −0.03**
	Speed‐adjusted partial correlation	*p* = 0.12	*p* = 0.73
		*ρ* = 0.29	*ρ* = −0.07
		95% CI = −0.11 to 0.63	95% CI = −0.47 to 0.39
Irregular terrain walking	Unadjusted correlation	***p* < 0.001**	***p* < 0.001**
		***ρ* = 0.59** ^a^	***ρ* = −0.59** ^a^
		**95% CI = 0.29–0.79**	**95% CI = −0.79 to −0.29**
	Speed‐adjusted partial correlation	*p* = 0.16	*p* = 0.12
		*ρ* = 0.28^a^	*ρ* = −0.30^a^
		95% CI = −0.02 to 0.62	95% CI = −0.57 to 0.1
Uphill walking	Unadjusted correlation	*p* = 0.06	***p* = 0.01**
		*ρ* = 0.35	***ρ* = −0.48**
		95% CI = −0.01 to 0.63	**95% CI = −0.72 to −0.15**
	Speed‐adjusted partial correlation	*p* = 0.87	*p* = 0.40
		*ρ* = −0.01	*ρ* = −0.16
		95% CI = −0.33 to 0.41	95% CI = −0.48 to 0.23
Downhill walking	Unadjusted correlation	***p* = 0.04**	***p* < 0.001**
		***ρ* = 0.38**	***ρ* = −0.58**
		**95% CI = 0.02–0.65**	**95% CI =** −**0.78 to** −**0.28**
	Speed‐adjusted partial correlation	*p* = 0.93	*p* = 0.18
		*ρ* = −0.02	*ρ* = −0.26
		95% CI = −0.30 to 0.36	95% CI = −0.53 to 0.12

*Note*: The bold formatting is used to highlight statistically significant correlations.

^a^ Twenty‐nine participants.

After adjustment for walking speed, none of the associations between Cw and NIHSS remained statistically significant across the walking tasks.

### 3.4 Association Between Cw and Barthel Index

Unadjusted analyses revealed significant negative associations between Cw and the Barthel Index, across several walking tasks. Low correlations were observed during overground walking (*ρ* = −0.41, *p* = 0.03), stair descent (*ρ* = −0.38, *p* = 0.04), and uphill walking (*ρ* = −0.48, *p* = 0.01). Moderate correlations were found during irregular terrain walking (*ρ* = −0.59, *p* < 0.001) (Figure 1B) and downhill walking (*ρ* = −0.58, *p* = 0.001). Overall, higher Cw values were associated with lower independence in ADL (Table 3).

After adjustment for walking speed, none of the associations between Cw and the Barthel Index remained statistically significant across the walking tasks.

## 4 Discussion

This study examined the associations between Cw and clinical impairment severity across several real‐life walking tasks in stroke survivors and whether these associations varied according to the task. A secondary objective was to explore the association between Cw and independence in ADL. Three main findings emerged. First, unadjusted analyses revealed significant associations between Cw, impairment severity, and independence in ADL across several walking tasks. Second, the strength of these associations varied across walking tasks, with stronger associations generally observed during more ecologically demanding conditions. Third, after adjustment for walking speed, none of the associations remained statistically significant, highlighting the central role of gait velocity in the relationship between Cw and clinical outcomes. These findings suggest that the clinical relevance of Cw may largely reflect the influence of walking speed rather than walking energetics per se.

### 4.1 Association Between Stroke Impairments and Cw Across Walking Tasks

Unadjusted analyses revealed low to moderate associations between stroke impairment severity, as assessed by the NIHSS, and Cw across several walking tasks, with the strongest association observed during irregular surface walking.

The relatively stronger unadjusted association observed during irregular surface walking likely reflects the increased mechanical and postural constraints imposed by this task rather than a specific independent effect of neurological impairment on Cw. Walking on irregular surfaces requires enhanced postural control, greater medio‐lateral stability, and more frequent corrective adjustments, resulting in increased neuromuscular demands and reduced dynamic stability in stroke survivors (Inui et al. 2023). Importantly, these additional postural and neuromuscular demands may contribute to greater reductions in walking speed, thereby indirectly contributing to higher Cw values.

Consistent with this interpretation, after adjustment for walking speed, none of the associations between NIHSS and Cw remained statistically significant, indicating walking speed may largely explain the observed relationships. These findings suggest that the association between impairment severity and Cw largely reflects gait velocity rather than isolated neurological deficits and that Cw should therefore not be interpreted independently of walking speed.

Because Cw is defined as the ratio between oxygen consumption and walking speed, individuals with greater impairment typically walk more slowly and therefore exhibit higher Cw values. This interpretation is consistent with previous findings by Reisman et al. (2009), who reported a strong negative correlation (*r* = −0.65, *p* = 0.006) between motor impairment severity assessed using the Fugl–Meyer scale and Cw during treadmill walking, highlighting the mediating role of walking speed. Although the NIHSS reflects overall neurological severity rather than isolated motor impairment, the consistency between our findings and those obtained using the Fugl–Meyer scale suggests that the observed associations are not solely dependent on the choice of clinical impairment scale.

### 4.2 Association Between Independence in ADL and Cw Across Walking Tasks

Unadjusted analyses revealed several negative associations between Cw and the Barthel Index, indicating that individuals with lower independence in ADL tended to walk with reduced gait efficiency. The strength of these associations varied across walking tasks, with stronger correlations observed during more demanding walking conditions, particularly those performed outdoors. These findings are consistent with previous work by Compagnat, Mandigout, et al. (2021), who reported a similar inverse association between Cw and the Barthel Index during a 6‐min walk test (*r* = −0.51, *p* < 0.01), and with Wert et al. (2013), who showed that higher Cw was associated with greater difficulty in mobility‐related ADL in older adults (*r* = −0.50, *p* < 0.001).

Notably, this task‐dependent pattern was not observed for all walking conditions. The lack of association observed during stair ascent further supports this interpretation. Stair climbing is a highly constrained task that primarily relies on concentric force production of the lower‐limb extensors and mechanical power output (Novak and Brouwer 2012). Unlike walking tasks that allow adaptive modulation of speed and movement strategies, stair ascent offers limited flexibility, thereby reducing the influence of independence in ADL on Cw. Although stair negotiation is included as an item in the Barthel Index, this scale does not capture the neuromuscular determinants underlying the metabolic demands of stair ascent. Consequently, tasks predominantly driven by muscular capacity rather than adaptive motor control may be less sensitive to differences in independence as reflected by ADL scales.

When walking speed was taken into account, none of the associations between Cw and independence in ADL remained statistically significant, suggesting that gait velocity largely explains the observed relationships. More autonomous individuals typically walk faster (Nindorera et al. 2022), which is generally associated with lower Cw values, and they also tend to engage in higher levels of daily walking activity (Fulk et al. 2017), supporting better cardiorespiratory fitness (Saunders et al. 2014) and, consequently, greater gait efficiency. Therefore, the clinical interpretation of Cw should be considered cautiously when walking speed is not simultaneously taken into account.

### 4.3 Study Limitations

Regarding the clinical characteristics of our cohort, the overall mild level of neurological impairment and the relatively preserved independence in ADL may have attenuated the strength of the observed associations, particularly due to a potential ceiling effect of the Barthel Index. Including individuals with more severe deficits could potentially reveal stronger relationships between Cw, impairment severity, and functional outcomes. In addition, impairment severity was assessed using the NIHSS, a global neurological scale that includes non‐motor domains and may not fully capture the motor impairments most directly related to walking energetics. Consequently, it may be less sensitive than motor‐specific measures, such as the Fugl–Meyer Assessment, for characterizing impairments associated with walking energetics. The use of walking aids by some participants may also have influenced walking speed, oxygen consumption, and consequently Cw measurements.

From a methodological perspective, the relatively small sample size may have limited statistical power, and the cross‐sectional design of the study precludes any inference regarding causal relationships. Multiple exploratory analyses conducted across several walking tasks may increase the risk of type I error. Consequently, the reported associations should be interpreted as exploratory and require confirmation in larger studies. Finally, although walking speed was adjusted for in the analyses, its inclusion in the calculation of Cw may complicate the interpretation of its independent contribution. Taken together, these limitations suggest that future studies involving larger and more heterogeneous stroke populations are needed to better characterize the determinants and functional consequences of an increased Cw after stroke.

### 4.4 Implications of Physiotherapy Practice

Cw showed several associations with the NIHSS across different walking tasks, suggesting that this parameter may reflect how residual neurological deficits are associated with increased energetic demands during walking. However, these associations were largely explained by walking speed, indicating that gait velocity represents a key mechanism linking impairment severity to increased Cw, rather than a direct effect of neurological deficits on Cw.

Regarding functional consequences, Cw demonstrated task‐dependent associations with independence in ADL that were more apparent in ecologically demanding walking conditions. This pattern highlights that physiological limitations may remain less visible during simple walking tasks but emerge when environmental constraints increase. From a clinical perspective, assessing Cw during ecologically challenging walking situations may provide complementary information regarding functional walking performance beyond standardized flat‐ground assessments. Nevertheless, given the central role of walking speed in the observed associations, Cw should not be interpreted independently of gait velocity.

## Funding

This research was supported by the Limoges University Hospital (CHU de Limoges) and the University of Limoges.

## Ethics Statement

The protocol was approved by the Ouest I Ethics Committee on July 12, 2022 (reference: 2022‐A01175‐38) and classified as a Type 2 interventional study according to French Jardé law.

## Consent

All participants provided written informed consent prior to inclusion.

## Conflicts of Interest

The authors declare no conflicts of interest.

## Permission to Reproduce Material From Other Sources

The authors have nothing to report.

## Data Availability

The datasets generated for this study are available on request to the corresponding author.
